# Tensor-Based Angle Estimation Approach for Strictly Noncircular Sources with Unknown Mutual Coupling in Bistatic MIMO Radar

**DOI:** 10.3390/s18092788

**Published:** 2018-08-24

**Authors:** Yuehao Guo, Xianpeng Wang, Wensi Wang, Mengxing Huang, Chong Shen, Chunjie Cao, Guoan Bi

**Affiliations:** 1 State Key Laboratory of Marine Resource Utilization in South China Sea and College of Information Science and Technology, Hainan University, Haikou 570228, China; gyhao1996@hainu.edu.cn (Y.G.); huangmx09@163.com (M.H.); chongshen@hainu.edu.cn (C.S.); chunjie_cao@126.com (C.C.); 2 School of Microelectronics, Beijing University of Technology, Beijing 100124, China; wensi.wang@bjut.edu.cn; 3 School of Electrical and Electronic Engineering, Nanyang Technological University, Singapore 639798, Singapore; egbi@ntu.edu.sg

**Keywords:** bistatic MIMO radar, angle estimation, strictly noncircular signal, mutual coupling, higher-order singular value decomposition

## Abstract

In the paper, the estimation of joint direction-of-departure (DOD) and direction-of-arrival (DOA) for strictly noncircular targets in multiple-input multiple-output (MIMO) radar with unknown mutual coupling is considered, and a tensor-based angle estimation method is proposed. In the proposed method, making use of the banded symmetric Toeplitz structure of the mutual coupling matrix, the influence of the unknown mutual coupling is removed in the tensor domain. Then, a special enhancement tensor is formulated to capture both the noncircularity and inherent multidimensional structure of strictly noncircular signals. After that, the higher-order singular value decomposition (HOSVD) technology is applied for estimating the tensor-based signal subspace. Finally, the direction-of-departure (DOD) and direction-of-arrival (DOA) estimation is obtained by utilizing the rotational invariance technique. Due to the use of both noncircularity and multidimensional structure of the detected signal, the algorithm in this paper has better angle estimation performance than other subspace-based algorithms. The experiment results verify that the method proposed has better angle estimation performance.

## 1. Introduction

Since the concept of multiple-input and multiple-output (MIMO) radar has been proposed in recent years, it has drawn a great attention of scholars in the field of radar research [1,2]. Since the MIMO radar transmits the orthogonal waveform and has different arrangement of transmit-receive array, MIMO radar can obtain both spatial diversity and waveform diversity at the same time [3]. For angle estimation, MIMO radar has better parameter estimation performance than the conventional phased array radar, especially for the estimation of joint direction-of-departure (DOD) and direction-of-arrival (DOA) [4]. The study of MIMO radar is mainly divided into two categories: (1) statistical MIMO radar [5,6], which can obtain the spatial diversity gain of both transmit and receive arrays for improving the detection and parameter estimation performance; and (2) collocated MIMO radar [7], which uses the orthogonality of the transmitted waveforms to form a large virtual array aperture for obtaining the corresponding waveform diversity gain. Thus, in the latter, the degrees of freedom (DOFs) are raised and the aperture of MIMO radar is enlarged. This literature focuses on the research of collocated MIMO radar.

In bistatic MIMO radar, the estimation of joint DOD and DOA is an important problem, and many popular algorithms have been proposed for this issue. Yan et al. [8] proposed the Capon method, and Gao et al. [9] proposed the multiple signal classification (MUSIC) method. These two methods belong to the spatial spectral method. A spatial spectral function is firstly constructed and then the angles can be estimated from the spatial spectrum. The advantage of the spatial spectrum methods is that they can realize the automatic matching between the DOD and DOA, and the accuracy of angle estimation is high. The disadvantage is that the computational complexity is large due to the two-dimensional spatial spectral searching. To remove the process of the spatial spectral searching, the estimation method of signal parameter via rotational invariance techniques (ESPRIT) is utilized to estimate the angles of targets in MIMO radar [10]. This method achieves the rotation invariance features for estimating DOD and DOA by dividing the virtual array into different subarrays, but this method cannot realize the automatic pairing between the transmit and receive angles. Then, the automatically-paired ESPRIT method is proposed in [11]. In Reference [12], the calculation procedure of ESPRIT algorithm is transformed into the real-valued domain by using unitary transformation, which reduces the computational complexity of ESPRIT algorithm without the performance loss. However, the algorithms mentioned above rely on the ideal transmit and receive arrays. Since the receive and transmit arrays cannot be accurately compensated, there exists the mutual coupling between the array elements [13]. In view of the mutual coupling of receive and transmit array elements in MIMO radar, several methods are proposed, such as MUSIC-like and ESPRIT-like algorithms [14,15]. The ESPRIT-like algorithm [14] utilizes the banded symmetric Toeplitz structure of the mutual coupling matrix in the uniform linear array to remove the effect of unknown mutual coupling, but it leads the loss of array aperture. Using inherent characteristics of the signals is considered as a possible way to compensate the aperture loss. Fortunately, the strictly noncircular signals, such as binary phase shift keying (BPSK) and M-ary amplitude shift keying (MASK), have been widely used in the field of communication and radar systems for aperture extension [16,17]. In view of using the noncircular characteristic of signal to improve the accuracy of DOA estimation, many algorithms have been developed [18,19,20]. The robust DOA estimation method with the unknown mutual coupling is investigated in [18], which takes the noncircular characteristic of the signal into account for eliminating the influence of mutual coupling. Then, the loss of array aperture is partly compensated by using the noncircular structure of these signals. On the other hand, all algorithms mentioned above need to stack the received data into a special structure matrix, which ignores the inherence multidimensional structure of signal. To utilize the inherent multidimensional structure of the signals, many methods have been developed [21,22,23,24,25]. A multi-SVD algorithm is developed to estimate DOD and DOA in MIMO radar [21], and the estimation performance is improved remarkably. Considering the mutual coupling in transmit and receive arrays, the subspace estimation method based on unitary tensor decomposition is introduced in [23]. The algorithm converts the tensor subspace into a new real-valued tensor through using the unitary transformation while eliminating the influence of mutual coupling. Then, the estimation of DOD and DOA is obtained by using tensor-based subspace, and it can achieve better angle estimation accuracy with lower computational burden. In addition, the DODs and DOAs can be estimated in the coexistence of mutual coupling and spatial colored noise [25]. According to the above analysis, these algorithms only utilize the noncircularity and inherent multidimensional structure of strictly noncircular signals separately in the case of unknown mutual coupling.

In this paper, we develop a tensor-based angle estimation scheme for strictly noncircular sources in the presence of unknown mutual coupling in bistatic MIMO radar. This method not only takes the multidimensional structure of the signals into account, but also uses the noncircular characteristics of the signals. Firstly, the proposed method uses the band symmetric Toeplitz structure of mutual coupling matrix to remove the influence of unknown mutual coupling. Then, a novel augmented tensor is constructed to utilize both the noncircularity and inherent multidimensional structure of strictly noncircular signals. Afterwards, the higher order SVD (HOSVD) technique of tensor decomposition is utilized to formulate a tensor-based signal subspace. Finally, the estimation of DODs and DOAs are obtained by utilizing the rotational invariance technique, where the DODs and DOAs are matched automatically. Due to the exploitation of inherent multidimensional structure and enlarged array aperture, the proposed method has better angle estimation performance than other algorithms in the presence of mutual coupling. The experiment results are carried out to prove the advantages of the proposed method.

The summary is as follows. Section 2 gives some basic concepts of tensor and the tensor-based signal model. The proposed method is developed in Section 3. Section 4 discusses the comments and specific analysis of the proposed method. Simulation results are given in Section 5. The conclusion of the proposed algorithm is given in Section 6.

Notation: ${\left(\cdot \right)}^{H}$, ${\left(\cdot \right)}^{T}$, ${\left(\cdot \right)}^{-1}$ and ${\left(\cdot \right)}^{*}$ indicate conjugate-transpose, transpose, inverse, and conjugate, respectively. ⊗ and ⊙ represent the Kronecker product and Khatri–Rao product, respectively. diag(⋅) is the diagonalization operation, and Toeplitz(r) means the symmetric Toeplitz matrix constructed by the vector r. vec(⋅) indicates the vectorization operation. arg(γ) represents the phase of γ, $I_{K}$ denotes a K×K identity matrix, $0_{L\times K}$ is the L×K zero matrix, and $\Gamma_{K}$ represents a matrix with ones on its anti-diagonal and zeros elsewhere.

## 2. Tensor Basic Concepts and Tensor-Based Signal Model

### 2.1. Tensor Basic Concepts

In this section, the basic concepts and operational rules of tensor are introduced. More information about tensor can be obtained from previous articles [26,27].

**Definition** **1.** *(Mode-n matrix unfolding). Let*$X\in {\mathbb{C}}^{I_{1}\times I_{2}\times \cdots \times I_{N}}$*be a tensor, and the mode-n matrix unfolding of a tensor*X*is indicated by*${\left[X\right]}_{\left(n\right)}$*. The*$\left(i_{1},i_{2},\ldots ,i_{N}\right)$*th element of*X*maps to the*$\left(i_{n},j\right)$*th element of*${\left[X\right]}_{n}$*, where*$j=1+\Sigma_{k=1,k\neq n}^{N}(i_{k}-1)J_{k}$*with*$J_{k}=\Pi_{m=1,m\neq n}^{k-1}I_{m}$.

**Definition** **2.** *(Mode-n tensor-matrix product). The mode-n product of*$X\in {\mathbb{C}}^{I_{1}\times I_{2}\times \cdots \times I_{N}}$*with a matrix*$A\in {\mathbb{C}}^{J_{n}\times I_{n}}$*is denoted by*$Y=X\times_{n}A$*, where*$Y\in {\mathbb{C}}^{I_{1}\times I_{2}\times \cdots I_{n-1}\times J_{n}\times I_{n+1}\times \cdots \times I_{N}}$*and*${\left[Y\right]}_{{}_{i_{1},\cdots ,i_{n-1},j_{n},j_{n+1,}\cdots i_{N}}}=$$\sum_{i_{n}=1}^{I_{n}}{\left[Y\right]}_{{}_{i_{1},\cdots ,i_{n},\cdots i_{N}}}\cdot {\left[A\right]}_{j_{n},i_{n}}$.

**Definition** **3.** *(The properties of the mode product). The mode-n tensor-matrix product satisfies the following properties*

(1) $$\left\{ \begin{array}{l} X\times_{n}A\times_{m}B=X\times_{m}A\times_{n}B,\;m\neq n\\ X\times_{n}A\times_{n}B=X\times_{n}(AB),\;m=n \end{array}\right.$$

(2) $${\left[X\times_{1}A_{1}\times_{2}A_{2}\times \cdots \times_{N}A_{N}\;\right]}_{\left(n\right)}=A_{n}\cdot {\left[X\right]}_{\left(n\right)}\cdot {\left[A_{N}⊗\ldots ⊗A_{n+1}⊗A_{n-1}⊗\ldots ⊗A_{2}⊗A_{1}\right]}^{T}$$

**Definition** **4.** *(Tensor decomposition). The HOSVD of a tensor*$X\in {\mathbb{C}}^{I_{1}\times I_{2}\times \cdots \times I_{N}}$*is given by*

(3) $$X=G\times_{1}U_{1}\times_{2}U_{2}\times_{3}\ldots \times_{N}U_{N}$$

*where*$G\in {\mathbb{C}}^{I_{1}\times I_{2}\times \cdot \cdot \cdot \times I_{N}}$*is the core tensor, and*$U_{n}\in {\mathbb{C}}^{I_{n}\times I_{n}}(n=1,2,3,\cdot \cdot \cdot ,N)$*is a unitary matrix, which is consist of the left singular vectors of*${\left[X\right]}_{\left(n\right)}$.

**Definition** **5.** *(Mode-n concatenation of two tensors). The mode-n concatenation of two tensors*$X\in {\mathbb{C}}^{I_{1}\times I_{2}\times \cdots \times I_{N}}$*and*$Y\in {\mathbb{C}}^{I_{1}\times I_{2}\times \cdots \times I_{N}}$*is denoted as*$F=[X{⊥}_{n}Y]$*, where*$F\in {\mathbb{C}}^{I_{1}\times I_{2},\cdots ,\times 2I_{N}\times ,\cdots \times I_{N}}$.

### 2.2. Tensor-Based Signal Model

Consider a narrowband bistatic MIMO radar, which consists of an M-element transmit array and an N-element receive array, both of which are composed of half-wavelength spaced uniform linear arrays (ULAs). At the transmit array, M transmit antennas emit M mutual orthogonal strictly noncircular signals, such as BPSK modulated signals. Assume that there are K independent Swerling I targets with low speed in the far field. For the transmit and the receive arrays, the transmit and receive angles of the K target are denoted as $\varphi_{k}$ and respectively. The reflected signals are collected by the receive antennas and dealt with matched filters formed by the transmitted orthogonal waveforms. Then, the output of the received signal can be expressed as [19,20,21].

(4) $$\bar{X}(t_{l})=A_{r}\sum (t_{l})A_{t}^{T}+N(t_{l}),\;l=1,2,\cdots ,L$$

where $\bar{X}(t_{l})\in {\mathbb{C}}^{N\times M}$ is the received data at the lth snapshots, $A_{r}=[a_{r}(\theta_{1}),a_{r}(\theta_{2}),\cdots ,a_{r}(\theta_{K})]\in {\mathbb{C}}^{N\times K}$ is the receive steering matrix consisting of receive steering vector $a_{r}(\theta_{k})=[1,\exp(j\pi \sin\theta_{k}),\cdots ,$$\exp(j\pi (N-1)\sin\theta_{k}){]}^{T}$, $A_{t}=[a_{t}(\varphi_{1}),a_{t}(\varphi_{2}),\cdots ,a_{t}(\varphi_{K})]\in {\mathbb{C}}^{M\times K}$ is the transmit steering matrix consisting of the transmit steering vector $a_{t}(\varphi_{k})={\left[1,\exp(j\pi \sin\varphi_{k}),\cdots ,\exp(j\pi (M-1)\sin\varphi_{k})\right]}^{T}$. $\sum (t_{l})=diag(s(t_{l}))\in {\mathbb{C}}^{K\times K}$ is the strictly noncircular signal data with $s(t_{l})={\left[s_{1}(t_{l}),s_{2}(t_{l}),\cdots ,s_{K}(t_{l})\right]}^{T}$ at the lth snapshots, and the noncircular signal vector $s(t_{l})$ satisfies with $s(t_{l})=\Delta s_{c}(t_{l})$ where $\Delta =diag([\exp(j\phi_{1}),\exp(j\phi_{2}),\cdots ,$$\exp(j\phi_{K})])$ with the arbitrary phases $\phi_{k}(k=1,2,\cdots ,K)$ is assumed to be different for each source, and $s_{c}(t_{l})=s_{c}^{*}(t_{l})$, $N(t_{l})\in {\mathbb{C}}^{N\times M}$ is the additional Gaussian white noise matrix.

On account of the radiation effect between the antenna elements, the effect of mutual coupling will be produced [13], and the mutual coupling between antenna elements of uniform linear array can be expressed as banding symmetric Toeplitz matrix, which is called mutual coupling matrix. The mutual coupling coefficient between two antennas in a ULA is inversely proportional to the distance between them, and the mutual coupling coefficient decreases with the increase of the distance, and vice versa [14]. Assume that there are P+1 nonzero mutual coupling coefficients for both transmit and receive arrays, and P is satisfied with min{M,N}>2P. Taking the influence of mutual coupling into account, the received signal in Equation (4) can be expressed as

(5a) $$X(t_{l})=[C_{r}A_{r}]\sum (t_{l}){[C_{t}A_{t}]\;}^{T}+N(t_{l})$$

where $C_{t}=\mathrm{toeplitz}([c_{t}^{T},0_{1\times (M-P-1)}])\in C^{M\times M}$ and $C_{r}=\mathrm{toeplitz}([c_{t}^{T},0_{1\times (N-P-1)}])\in C^{N\times N}$ are the mutual coupling matrices with $c_{t}=[c_{t0},c_{t1},\cdot \cdot \cdot ,c_{tP}]$ and $c_{r}=[c_{r0},c_{r1},\cdot \cdot \cdot ,c_{rP}]$, $c_{ip}(i=r,t;p=0,1,2,\cdot \cdot \cdot ,P)$ is the P+1 nonzero mutual coupling coefficients, which satisfy with $0<|c_{ip}|<,\cdot \cdot \cdot ,<|c_{i1}|<|c_{i0}|=1$. The mutual coupling matrices have the banded symmetric Toeplitz structure. In other words, the mutual coupling matrix $C_{i}(i=r,t)$ can be expressed as

(5b) $$C_{i}=\left[\begin{array}{lllllllll} c_{i0} & c_{i1} & \cdots & c_{ip} & & & & & \\ c_{i1} & c_{i0} & c_{i1} & \cdots & c_{ip} & & & 0 & \\ \vdots & \ddots & \ddots & \ddots & \vdots & \ddots & & & \\ c_{ip} & \cdots & c_{i1} & c_{i0} & c_{i1} & \cdots & c_{ip} & & \\ & \ddots & \vdots & \ddots & \ddots & \ddots & & \ddots & \\ & & c_{ip} & \cdots & c_{i1} & c_{i0} & c_{i1} & \cdots & c_{ip}\\ & & & \ddots & \vdots & \ddots & \ddots & \ddots & \vdots \\ & & 0 & & c_{ip} & \cdots & c_{i1} & c_{i0} & c_{i1}\\ & & & & & c_{ip} & \cdots & c_{i1} & c_{i0} \end{array}\right]$$

According to the basic concepts and operational rules of tensor, the received signal $X(t_{l})(l=1,2,\cdots ,L)$ can be seen as different slices of a third-order tensor along the direction of snapshot (the third-dimension). By collecting L snapshots, a third-order tensor data $X\in {\mathbb{C}}^{N\times M\times L}$ is formed as

(6) $$[X_{:,:,l\;}]=X(t_{l}),l=1,2,\cdots ,L$$

where $\left[X_{:,:,l}\right]$ is the lth slice of the tensor along the third-dimension. According to the definition of Mode-n matrix unfolding, the relationship between the tensor-based data model and matrix-based data model is expressed as

(7) $${[X]\;}_{\left(3\right)}^{T}=[{\bar{A}}_{t}⊙{\bar{A}}_{r}]S+N=[{\bar{A}}_{t}⊙{\bar{A}}_{r}]\Delta S_{c}+N$$

where ${\bar{A}}_{t}=C_{t}A_{t}$, ${\bar{A}}_{r}=C_{r}A_{r}$ is the transmit-receive steering matrix, $S=[s(t_{1}),s(t_{2}),\cdots ,s(t_{L})]\in {\mathbb{C}}^{K\times L}$ is the signal matrix, and $S_{c}=[s_{c}(t_{l}),s_{c}(t_{2}),\cdots ,s_{c}(t_{L})]\in {\mathbb{R}}^{K\times L}$ satisfies with $S_{c}=S_{c}^{*}$. $N=[\mathrm{vec}(N(t_{l})),$ $\mathrm{vec}(N(t_{l})),\cdots ,\mathrm{vec}(N(t_{L}))]\in {\mathbb{C}}^{MN\times L}$ is the noise matrix.

## 3. Tensor-Based Angle Estimation Method with Unknown Mutual Coupling

In this section, a tensor-based angle estimation algorithm is investigated for capturing the noncircularity and inherent multidimensional structure of the received signal to improve the accuracy of angle estimation in the case of unknown mutual coupling.

### 3.1. Mutual Coupling Elimination

In Equation (7), the mutual coupling affects the transmit direction matrix ${\bar{A}}_{t}$ and the receive direction matrix ${\bar{A}}_{r}$, therefore the Vandermonde structure of ${\bar{A}}_{t}$ and ${\bar{A}}_{r}$ are destroyed. Fortunately, the mutual coupling matrices are banded symmetric Toeplitz. It can extract two sub-matrices from transmit and receive direction matrices to elimination the influence of mutual coupling. By defining two choice matrices

(8) $$\begin{array}{l} J_{1}=[0_{(N-2P)\times P\;},I_{\left(N-2P\right)},0_{(N-2P)\times P}]\\ J_{2}=[0_{(M-2P)\times P},I_{\left(M-2P\right)},0_{(M-2P)\times P}] \end{array}$$

based on the characteristics of the mutual coupling matrix, we have

(9) $$\left\{ \begin{array}{l} {\hat{a}}_{r}(\theta_{k})=J_{1}{\bar{a}}_{r}(\theta_{k})=\beta_{rk\;}{\tilde{a}}_{r}(\theta_{k})\\ {\hat{a}}_{t}(\varphi_{k})=J_{2}{\bar{a}}_{t}(\varphi_{k})=\beta_{tk}{\tilde{a}}_{t}(\varphi_{k}) \end{array}\right.$$

where $\beta_{tk}=1+\sum_{p=1}^{p}2c_{tp}\cos(p\pi \sin\varphi_{k})$, $\beta_{rk}=1+\sum_{p=1}^{p}2c_{rp}\cos(p\pi \sin\theta_{k})$. ${\bar{a}}_{t}(\varphi_{k})$ and ${\bar{a}}_{r}(\theta_{k})$ are the transmit and receive steering vectors with mutual coupling, respectively. ${\tilde{a}}_{r}(\theta_{k})$ and ${\tilde{a}}_{t}(\varphi_{k})$ are the column vectors of the first $\tilde{N}=N-2P$ and $\tilde{M}=M-2P$ elements of $a_{r}(\theta_{k})$ and $a_{t}(\varphi_{k})$. It can be clearly seen from Equation (9) that parameters $\beta_{tk}$ and $\beta_{rk}$ are constant for each target, which means that the direction matrices ${\hat{A}}_{r}(\theta )=[{\hat{a}}_{r}(\theta_{1}),{\hat{a}}_{r}(\theta_{2}),\cdot \cdot \cdot ,{\hat{a}}_{r}(\theta_{K})]$ and ${\hat{A}}_{t}(\theta )=[{\hat{a}}_{t}(\theta_{1}),{\hat{a}}_{t}(\theta_{2}),\cdot \cdot \cdot ,{\hat{a}}_{t}(\theta_{K})]$ are the Vandermonde matrices [23]. Therefore, the effect of mutual coupling is removed after. The procedure of decoupling in Equation (9) can be extended to the tensor domain in Equation (7):

(10) $$\hat{X}=X_{\times 1\;}J_{1\times 2}J_{2}=I_{K\times 1}{\hat{A}}_{r\times 2}{\hat{A}}_{t\times 3}S+\hat{N}$$

where $\hat{N}=N_{\times 1}J_{1\times 2}J_{2}$ is a part of N, the tensor noise $\hat{N}$ has the same properties as the N.

Then, according to the definition Mode-n matrix unfolding, the mode-3 matrix unfolding of $\hat{X}\in {\mathbb{C}}^{2\tilde{N}\times \tilde{M}\times L}$ is written as

(11) $${[\hat{X}]\;}_{\left(3\right)}^{T}=[{\hat{A}}_{t}⊙{\hat{A}}_{r}]S+\hat{N}=[{\hat{A}}_{t}⊙{\hat{A}}_{r}]\Delta S_{c}+\hat{N}$$

From the above analysis, we can see that ${\hat{A}}_{t}$ and ${\hat{A}}_{r}$ have Vandermonde structure. It can be shown that the mutual coupling effect has been removed in the tensor domain.

### 3.2. Tensor Augmentation and Signal Subspace Estimation

To utilize the noncircular property of the signal in the tensor domain, a special augmented tensor is constructed by the tensor-based forward and backward smoothing technique:

(12) $$Y=[\hat{X}{⊥}_{1}({\hat{X}}^{*}\times_{1}\Gamma_{\tilde{N}}\times_{2}\Gamma_{\tilde{M}})]\;$$

Then, according to the definition Mode-n matrix unfolding, the mode-3 matrix unfolding of $Y\in {\mathbb{C}}^{2\tilde{N}\times \tilde{M}\times L}$ is written as

(13) $${[Y]\;}_{\left(3\right)}^{T}=[{\hat{A}}_{t}⊙{\overset{‿}{A}}_{r}]S_{c}+\hat{N}$$

where ${\overset{‿}{A}}_{r}={\left[{\left({\hat{A}}_{r}^{1}\right)}^{T},{\left({\hat{A}}_{r}^{2}\right)}^{T}\right]}^{T}\in {\mathbb{C}}^{2\tilde{M}\tilde{N}\times K}$ denotes the extended steering matrix, where ${\hat{A}}_{r}^{1}={\hat{A}}_{r}\Delta$ and ${\hat{A}}_{r}^{2}={\hat{A}}_{r}\Delta^{*}D_{t}D_{r}$ with $D_{t}=\mathrm{diag}([\exp(-j\pi (\tilde{M}-1)\sin\varphi_{1}),\exp(-j\pi (\tilde{M}-1)\sin\varphi_{2}),\cdots ,\exp(-j\pi (\tilde{M}-1)\sin\varphi_{K})])$ and $D_{r}=\mathrm{diag}([\exp(-j\pi (\tilde{N}-1)\sin\theta_{1}),\exp(-j\pi (\tilde{N}-1)\sin\theta_{2}),\cdots ,\exp(-j\pi (\tilde{N}-1)\sin\theta_{K})])$, $\hat{N}$ is the modified noise matrix. It is easy to know from Equation (13) that the available array aperture is twice the model in Equation (11). It can be found that the augmented tensor Y not only considers the multidimensional structure of tensor, but also captures the noncircularity of the signal and enlarges the virtual aperture of the array. Thus, a better performance of parameter estimation is expected to be achieved in the proposed method. Based on the augmented tensor in Equation (12), the HOSVD method is applied to the augmented tensor Y:

(14) $$Y=G\times_{1}E_{1}\times_{2}E_{2}\times_{3}E_{3}$$

where $E_{1}\in {\mathbb{C}}^{2\tilde{N}\times 2\tilde{N}}$, $E_{2}\in {\mathbb{C}}^{\tilde{M}\times \tilde{M}}$ and $E_{3}\in {\mathbb{C}}^{L\times L}$ are unitary matrices, which are made up of the left singular of the mode-n(n=1,2,3) of matrix unfolding of Y as ${\left[Y\right]}_{\left(n\right)}=E_{n}\Lambda_{n}V_{n}^{H}$, respectively. $G\in {\mathbb{C}}^{2\tilde{N}\times \tilde{M}\times L}$ represents the core tensor. Because there are K sources, Y is rank-K tensor. Then, a subspace tensor is achieved by using the truncated HOSVD of Y, which is shown as

(15) $$Y_{s}=G_{s}\times_{1}E_{s1\;}\times_{2}E_{s2}$$

where $E_{sn}(n=1,2,3)$ is made up of the column vectors of $E_{n}$ corresponding to the largest K singular values, and $G_{s}=Y\times_{1}E_{s1}^{H}\times_{2}E_{s2}^{H}\times_{3}E_{3s}^{H}$ denotes the signal component of G. Then, according to the definition of Mode-n tensor-matrix product, substituting $G_{s}$ into Equation (15) yields

(16) $$Y_{s}=Y\times_{1}(E_{s1\;}E_{s1}^{H})\times_{2}(E_{s2}E_{s2}^{H})\times_{3}E_{3s}^{H}$$

Then, the tensor-based signal subspace is given by using the mode-3 matrix unfolding of $Y_{s}$, and according to the properties of the mode product, the tensor-based signal subspace is shown as

(17) $${\bar{U}}_{s}={[Y_{s}]\;}_{\left(3\right)}^{T}=(E_{s2}E_{s2}^{H}⊗E_{s1}E_{s1}^{H}){\left[Y_{s}\right]}_{\left(3\right)}E_{3s}^{*}$$

After using some simplification in [21,23,24], the tensor based signal subspace is written as

(18) $${\bar{U}}_{s}=(E_{s2\;}E_{s2}^{H}⊗E_{s1}E_{s1}^{H})U_{s}$$

where $U_{s}$ is the signal subspace of ${\left[Y_{s}\right]}_{\left(3\right)}$, which can be estimated by truncating SVD of ${\left[Y_{s}\right]}_{\left(3\right)}$ as ${\left[Y_{s}\right]}_{\left(3\right)}\approx U_{s}\Lambda_{s}V_{s}^{H}$. According to Equation (18), it is shown that the ${\bar{U}}_{s}$ and $U_{s}$ span to the same subspace, which means that the tensor based signal subspace ${\bar{U}}_{s}$ and augmented steering matrix $\overset{‿}{A}={\hat{A}}_{t}⊙{\overset{‿}{A}}_{r}$ also span to the same subspace. Thus, there is a nonsingular matrix T satisfied with ${\bar{U}}_{s}=\overset{‿}{A}T$, and the estimation of DODs and DOAs can be achieved from this tensor-based signal subspace.

### 3.3. Joint DOD and DOA Estimation

Noting that ${\hat{A}}_{t}$ has Vandermonde structure, there exists the following rotation invariance equation [10,11]

(19) $$\prod_{2}\overset{‿}{A}=\prod_{1}\overset{‿}{A}\Phi_{t}\;$$

where $\Phi_{t}=\mathrm{diag}([\exp(j\pi \sin\varphi_{1}),\exp(j\pi \sin\varphi_{2}),\cdots ,\exp(j\pi \sin\varphi_{k})])$ is s rotational invariance factor matrix that contains the desired information of DOAs. $\prod_{1}=J_{3}⊗I_{2\tilde{N}}$ and $\prod_{2}=J_{4}⊗I_{2\tilde{N}}$ are selection matrices with $J_{3}=[I_{\tilde{M}-1},O_{(\tilde{M}-1)\times 1}]$ and $J_{4}=[O_{(\tilde{M}-1)\times 1},I_{\tilde{M}-1}]$, respectively. Simultaneously, both ${\hat{A}}_{r}^{1}$ and ${\hat{A}}_{r}^{2}$ have Vandermonde-like structures in ${\overset{‿}{A}}_{r}$. There is another rotation invariance equation:

(20) $$\prod_{4}\overset{‿}{A}=\prod_{3}\overset{‿}{A}\Phi_{r}\;$$

where $\Phi_{r}=\mathrm{diag}([\exp(j\pi \sin\theta_{1}),\exp(j\pi \sin\theta_{2}),\cdots ,\exp(j\pi \sin\theta_{k})])$ contains the desired information of DODs. $\prod_{3}=I_{2\tilde{M}}⊗J_{5}$ and $\prod_{4}=I_{2\tilde{M}}⊗J_{6}$ are the selection matrices with $J_{5}=[I_{\tilde{N}-1},O_{(\tilde{N}-1)\times 1}]$ and $J_{6}=[O_{(\tilde{N}-1)\times 1},I_{\tilde{N}-1}]$, respectively. Utilizing the relationship between the augmented steering matrix and tensor-based signal subspace shown as ${\bar{U}}_{s}=\overset{‿}{A}T$, the following rotational invariance property can be achieved, which is shown as

(21) $$\prod_{2}{\bar{U}}_{s}=\prod_{1}{\bar{U}}_{s}\Psi_{t}\;\prod_{4}{\bar{U}}_{s}=\prod_{3}{\bar{U}}_{s}\Psi_{r}\;$$

where $\Psi_{t}=T\Phi_{t}T^{-1}$ and $\Psi_{r}=T\Phi_{r}T^{-1}$. The least squares (LS) or the total least squares (TLS) technique is applied to Equation (21) for estimating $\Psi_{t}$ and $\Psi_{r}$. Then, the estimation of $\Phi_{t}$ can be obtained through the EVD of $\Psi_{t}$, and supposing $\bar{T}$ be the eigenvector matrix of $\Psi_{t}$. To achieve the estimation of the DOAs paired with the estimated DODs, calculate the $\Phi_{r}$ via $\bar{T}\Psi_{r}{\bar{T}}^{-1}$. Finally, the DODs and DOAs are derived as

(22) $${\hat{\varphi }}_{p}=\arcsin[\arg(u_{k})/\pi ]\;{\hat{\theta }}_{p}=\arcsin[\arg(v_{k})/\pi ]\;$$

where $u_{k}$ and $v_{k}$ are the kth diagonal elements of $\Phi_{t}$ and $\Phi_{r}$, respectively.

## 4. Remarks and Algorithm Analysis

### 4.1. Related Remarks

**Remark** **1.** *If a signal has only the in-phase component and the orthogonal component is zero, the signal is called a noncircular signal. The difference between a noncircular signal and a circular signal is whether the elliptic covariance is equal to zero. For a noncircular signal, the elliptic covariance is not equal to zero, which means that more information can be used. Therefore, the number of available array elements can be increased by reconstructing the receiving data matrix of noncircular signals.*

**Remark** **2.** *The methods proposed in [14,15] are also based on Equation (7), but these two methods ignore the multidimensional and noncircular characteristics of the measurement tensor*Y, *so the estimation performance is not satisfactory. Additionally, the methods proposed in [19,22] utilize the noncircular characteristics and multidimensional structure of signals respectively. However, both methods are completely invalid under the condition of mutual coupling.*

**Remark** **3.** *For the tensor-based signal model in Equation (6), the method in [23] investigates the way to remove the influence of mutual coupling in tensor domain for improving the performance. On the other hand, based on the matrix-based signal model in Equation (7), the noncircularity of signals is utilized to enlarge the array aperture after removing the effect of mutual coupling in [18]. It has been shown that the existing methods consider the noncircularity and inherent multidimensional structure of strictly noncircular signals separately with unknown mutual coupling. However, the proposed algorithm utilizes the noncircularity and the inherent multidimensional structure simultaneously, which results in more accurate signal subspace estimation and excellent angle estimation performance. The experiment results will show its advantage.*

### 4.2. Computation Complexity

According to Golub et al. [28], it is known that for a M×N dimensional matrix, the K rank truncating SVD decomposition requires O(MNK) complexity. The computational complexity of the algorithm proposed in this paper is mostly concentrated on the high order singular value decomposition of the tensor Y. In other words, the three-dimensional SVD decomposition is used for the tensor Y, so the corresponding computational complexity is $O(6\tilde{M}\tilde{N}K)$. On the other hand, the computational complexity of the Tensor unitary ESPRIT algorithm in [23] is $O(\tilde{M}\tilde{N}K3/4)$. Thus, the algorithm proposed in this paper has higher computational burden than Tensor unitary ESPRIT algorithm, but it has superior angle estimation performance.

## 5. Simulation Results

In this part, some numerical experiments are carried out to prove that the proposed algorithm has superior angle estimation performance. ESPRIT-like algorithm [14], Tensor unitary ESPRIT algorithm [23] and Cramer–Rao bound (CRB) [14] were compared with the proposed method. In these simulations, the bistatic MIMO radar is made up of M=8 transmit antennas and N=10 receive antennas, both of which are composed of half-wavelength spaced uniform linear arrays (ULAs). Unless stated otherwise, it was assumed that there are K=3 uncorrelated targets, located at $\left(\varphi_{1},\theta_{1})=(5^{{}^{\circ}},-8^{{}^{\circ}}\right)$, $\left(\varphi_{2},\theta_{2})=(-5^{{}^{\circ}},15^{{}^{\circ}}\right)$ and $\left(\varphi_{3},\theta_{3})=(10^{{}^{\circ}},-5^{{}^{\circ}}\right)$. The root mean square error (RMSE) was utilized to achieve the evaluation of angle estimation performance, which is expressed as

(23) $$\mathrm{RMSE}=\sqrt{\frac{1}{2\mathrm{QK}}\sum_{k=1}^{K}\sum_{i=1}^{Q}\left[{\left({\hat{\varphi }}_{k,i}-\varphi_{k}\right)}^{2}+{\left({\hat{\theta }}_{k,i}-\theta_{k}\right)}^{2}\right]}$$

where ${\hat{\varphi }}_{k,i}$ and ${\hat{\theta }}_{k,i}$ are the estimation of DOD $\varphi_{k}$ and DOA $\theta_{k}$ for the *i*th Monte Carlo trial, respectively. the total number of Monte Carlo trials was assumed as Q, and Q=500 was used in the following simulations. The other parameter is the probability of the successful detection (PSD) expressed as PSD=(D/Q)×100%, where D represents the total number of successful times and a successful trial requires the absolute error of all the experiment results are smaller than $\min[{\left({\hat{\varphi }}_{k}-\varphi_{k}\right)}_{k=1}^{K},{\left({\hat{\theta }}_{k}-\theta_{k}\right)}_{k=1}^{K}]$. For the mutual coupling parameters, there are two cases: (1) P=1 with $c_{t}=[1,0.1185+j0.058]$ and $c_{r}=[1,0.1520+j0.0248]$; and (2) P=2 with $c_{t}=[1,0.72+j0.03,0.18+j0.072]$ and $c_{r}=[1,0.58+j0.0145,0.13+j0.0482]$.

**Figure 1:**
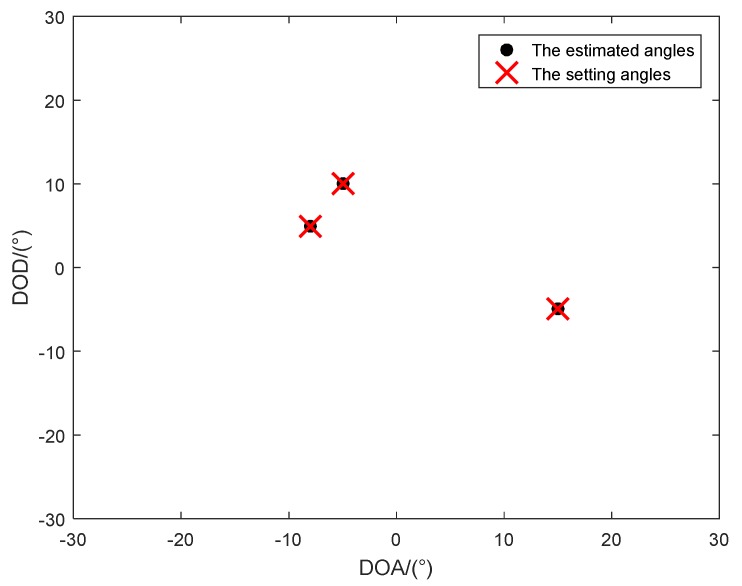
Estimation results of the proposed method with SNR = 0 dB (*K* = 3 targets, *P* = 1).

**Figure 2:**
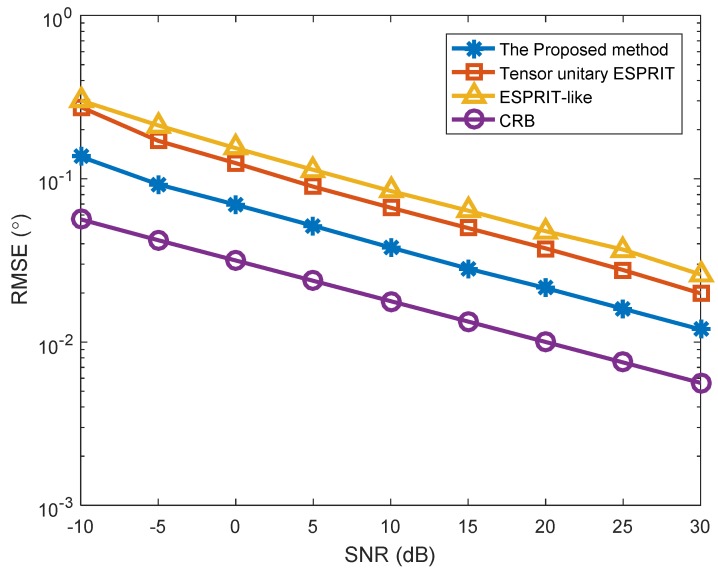
RMSE versus SNR with different algorithms (*K* = 3 targets, *P* = 1).

**Figure 3:**
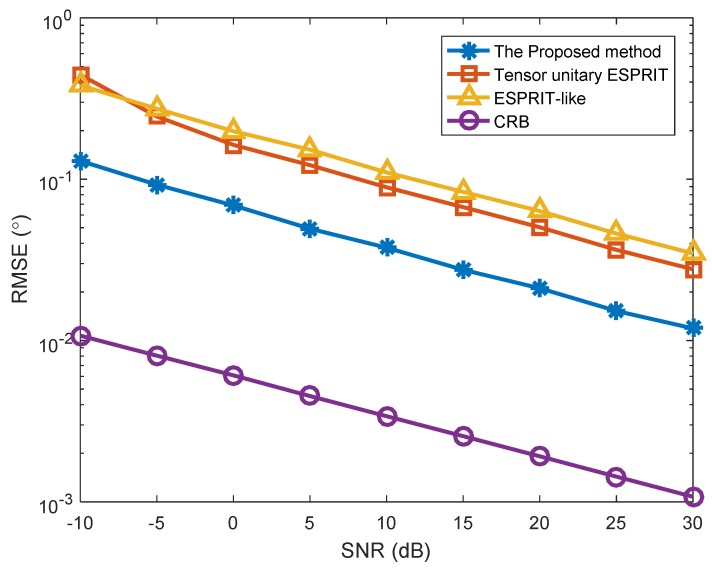
RMSE versus SNR with different algorithms (*K* = 3 targets, *P* = 2).

In the first simulation, we investigated the estimation results of the proposed method, and the SNR versus RMSE in two different situations (Figure 1, Figure 2 and Figure 3). The number of snapshots is L=100. Figure 1 shows the estimation results of the proposed algorithm with SNR = 0 dB in Case (1). We can clearly see that DODs and DOAs were correctly identified and matched accurately, which verifies the validity of the proposed algorithm. Figure 2 depicts the RMSE versus SNR with different methods in Case (1). At the same time, Figure 3 depicts the RMSE versus SNR in Case (2). In Figure 2, the angle estimation performance of the proposed method is clearly superior to the Tensor unitary ESPRIT algorithm and the ESPRIT-like algorithm, and the performance of the proposed algorithm is close to the Cramer–Rao bound (CRB). That is because the proposed method not uses the multidimensional structure of the signal, but also utilizes the noncircularity characteristics. Other methods only consider the noncircular structure or tensor multidimensional structure. In addition, the performance of the Tensor unitary ESPRIT method is better than the ESPRIT-like method, because the Tensor unitary ESPRIT method considers the multidimensional structure of the signal and obtains superior estimation performance with unknown mutual coupling. Similar conclusions can be achieved from Figure 3, which means that the proposed method can obtain superior performance in both cases.

**Figure 4:**
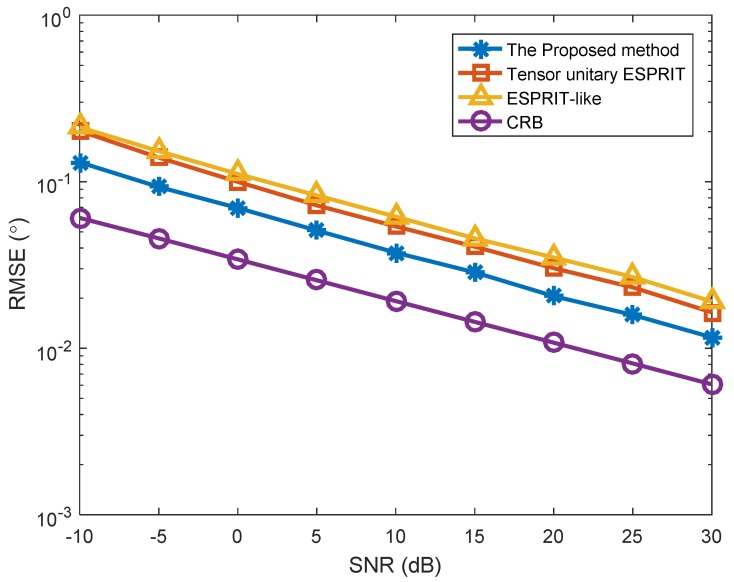
RMSE versus SNR with different algorithms (*K* = 2 targets, *P* = 1).

**Figure 5:**
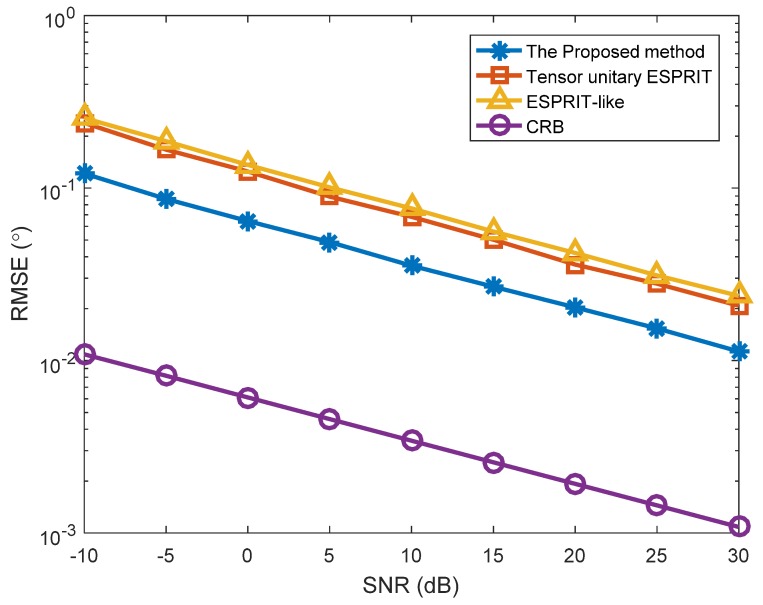
RMSE versus SNR with different algorithms (*K* = 2 targets, *P* = 2).

In the second simulation, we analyzed the angle estimation performance of different algorithms in the presence of K=2 targets, where the two targets are located at $\left(\varphi_{1},\theta_{1})=(10^{{}^{\circ}},-5^{{}^{\circ}}\right)$ and $\left(\varphi_{2},\theta_{2})=(5^{{}^{\circ}},0^{{}^{\circ}}\right)$. The number of snapshots was L=100 and the mutual coupling in Figure 4 is set as Case (1). At the same time, Figure 5 depicts the RMSE versus SNR in Case (2). Figure 4 depicts the RMSE versus SNR with different algorithms for two targets. In Figure 4, the performance of our proposed method is still superior to that of Tensor unitary ESPRIT method and ESPRIT-like method. In addition, the estimation performance of Tensor unitary ESPRIT method is superior to that of ESPRIT-like method. Similar conclusions can be achieved from Figure 5, which means that the proposed method can obtain superior performance in both cases.

**Figure 6:**
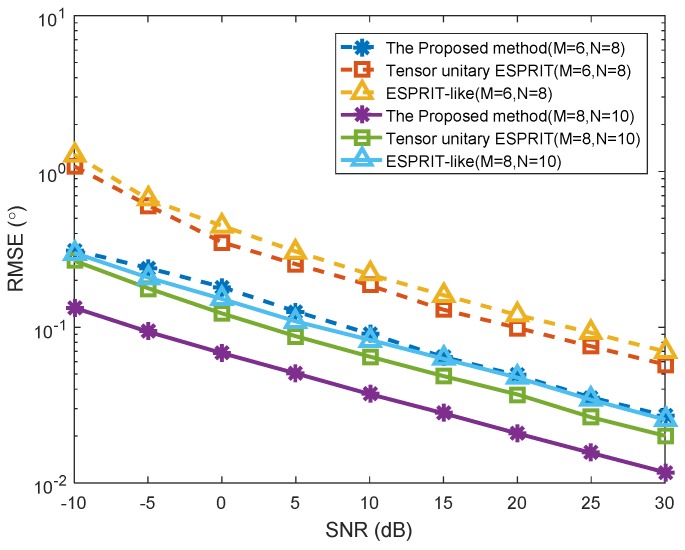
RMSE versus SNR with different transmit–receive array configurations (*K* = 3 targets, *P* = 1).

The third simulation indicates the RMSE versus SNR of different transmit–receive array configurations for K=3 targets in Case (1). As shown in Figure 6, the angle estimation performance of all the three algorithms improved with the increasing of the elements of transmit and receive arrays, in which the configuration of transmit–receive array is M=6,N=8 and M=8,N=10, respectively. The main reason is that more spatial diversity gain of MIMO radar was obtained with more transmit and receive arrays. Finally, the spatial resolution of the proposed method is improved.

**Figure 7:**
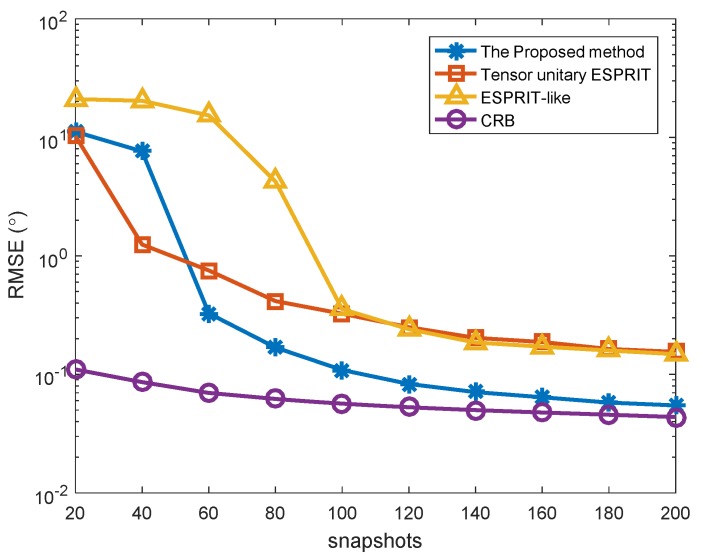
RMSE versus the number of snapshots with different algorithms (*K* = 3 targets, *P* = 1).

The fourth simulation describes the RMSE versus snapshots of different algorithms for K=3 targets, where SNR = 0 dB and the mutual coupling is set as Case (1). In Figure 7, the performance of all algorithms improved with more snapshots. The performance of our proposed algorithm is superior to that of several other methods in general, but the performance of the Tensor unitary ESPRIT method is slightly better than the proposed method under very low snapshots, which is because the Tensor unitary ESPRIT method increases the number of snapshots effectively by spatial smoothing. When the number of snapshots is greater than a specific threshold, the performance of the proposed method is superior to that of Tensor unitary ESPRIT method and ESPRIT-like method, and is very close to CRB. In addition, the performance of the Tensor unitary ESPRIT method is close to the performance of the ESPRIT-like method under the condition of large snapshot number, but it is still inferior to the proposed method.

**Figure 8:**
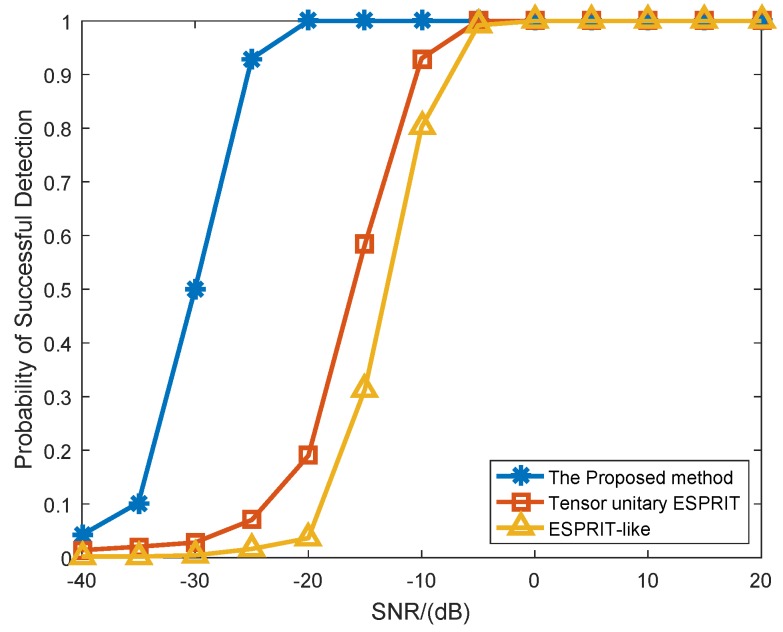
Probability of successful detection versus SNR (*K* = 3 targets, *P* = 1).

The fifth simulation depicts the probability of successful detection of several algorithms versus SNR for K=3 targets, where the number of snapshots was L=100 and the mutual coupling is set as Case (1). In Figure 8, all algorithms can achieve 100% accuracy at high SNR region, but the accuracy of the proposed method can reach 100% faster at certain SNR. In other words, in the same case of SNR, the proposed algorithm has a higher PSD than other algorithms. That is mainly because the proposed algorithm can reasonably utilize the noncircular characteristics and multidimensional structure characteristics of signals to promote the performance of angle estimation.

## 6. Conclusions

In this paper, a tensor-based angle estimation approach is proposed for strictly noncircular signals with unknown mutual coupling in MIMO radar. The proposed algorithm can capture both noncircularity and multidimensional structure of signals via formulating a novel augmented tensor. Meanwhile, it can remove the influence of unknown mutual coupling in the tensor domain. As a result, the proposed method has superior angle estimation to the existing subspace-based methods. The advantage of the proposed algorithm is clearly demonstrated using numerical experiments.
